# Impact of microclimatic conditions and resource availability on spring and autumn phenology of temperate tree seedlings

**DOI:** 10.1111/nph.17606

**Published:** 2021-08-16

**Authors:** Yann Vitasse, Frederik Baumgarten, Constantin M. Zohner, Rungnapa Kaewthongrach, Yongshuo H. Fu, Manuel G. Walde, Barbara Moser

**Affiliations:** ^1^ WSL Swiss Federal Institute for Forest, Snow and Landscape Research Birmensdorf CH‐8903 Switzerland; ^2^ Institute of Integrative Biology ETH Zürich (Swiss Federal Institute of Technology) Zürich CH‐8092 Switzerland; ^3^ Geo‐Informatics and Space Technology Development Agency (GISTDA) Chonburi 20230 Thailand; ^4^ College of Water Sciences Beijing Normal University Beijing 100875 China

**Keywords:** bud albedo, budburst, drought, environmental stressors, leaf senescence, light, microclimate, phenology

## Abstract

- Microclimatic effects (light, temperature) are often neglected in phenological studies and little information is known about the impact of resource availability (nutrient and water) on tree’s phenological cycles.
- Here we experimentally studied spring and autumn phenology in four temperate trees in response to changes in bud albedo (white‐painted vs black‐painted buds), light conditions (nonshaded vs *c*. 70% shaded), water availability (irrigated, control and reduced precipitation) and nutrients (low vs high availability).
- We found that higher bud albedo or shade delayed budburst (up to +12 d), indicating that temperature is sensed locally within each bud. Leaf senescence was delayed by high nutrient availability (up to +7 d) and shade conditions (up to +39 d) in all species, except oak. Autumn phenological responses to summer droughts depended on species, with a delay for cherry (+7 d) and an advance for beech (−7 d).
- The strong phenological effects of bud albedo and light exposure reveal an important role of microclimatic variation on phenology. In addition to the temperature and photoperiod effects, our results suggest a tight interplay between source and sink processes in regulating the end of the seasonal vegetation cycle, which can be largely influenced by resource availability (light, water and nutrients).

## Introduction

The phenological responses of plants to environmental cues play a prominent role in shaping species’ distribution ranges (Chuine & Beaubien, 2001; Körner *et al*., 2016) and Earth’s climate (Richardson *et al*., 2013). Over recent years, a profusion of studies based on ground observations or remote sensing have documented phenological shifts in response to global warming, consistently showing earlier occurrence of spring phenophases, such as leaf‐out or flowering, and, in some cases, later autumn phenophases, such as fruit maturation or leaf senescence (Garonna *et al*., 2016; Piao *et al*., 2019). Changes in phenology have a major impact on the global carbon balance, and earlier leaf‐out timing has been shown to compensate for the increasing carbon loss in summer due to more severe and prolonged drought (Wolf *et al*., 2016) but earlier phenology may also accelerate and amplify drought in early summer as plants begin to take up water earlier (Ma *et al*., 2016; Xu *et al*., 2020; Meier *et al*., 2021). For these reasons, increasing efforts have been made to include phenological models in global models of species distribution and forest carbon balance (Delpierre *et al*., 2016; Zohner *et al*., 2020). Phenological models are still however unable to accurately predict the progression of winter dormancy, which is essential for predicting the beginning of bud development in spring (Basler, 2016; Chuine *et al*., 2016; Wang *et al*., 2020), as well as the time of leaf senescence in autumn (Liu *et al*., 2020). A striking example is that simplistic spring phenology models that ignore chilling and photoperiod often perform similarly compared with more complex phenological models that include these cues (Fu *et al*., 2012; Basler, 2016). This contrasts with numerous experimental observations of temperate and boreal perennial plants, which have long shown that chilling and photoperiod play a significant role in dormancy release and bud development (e.g. Coville, 1920; Wareing, 1953; Murray *et al*., 1989; Heide, 1993; Rousi & Pusenius, 2005; Viherä‐Aarnio *et al*., 2006).

A major limitation of plant physiological and phenological studies carried out under natural conditions is that they usually use the temperature recorded at standard weather stations as an approximation of the temperature perceived by the plant. The microclimate in forests or near buds can largely deviate from 2‐m height air temperature (for example buds of seedlings and saplings are close to the ground whereas buds of adult trees can be at a height of 30 m with more exposure to wind and solar radiation) and even more from standard air temperature measured outside of the forest (De Frenne *et al*., 2019). Yet, microclimatic conditions have a huge effect on plant performance, and changes in microclimate have even been shown to outweigh macroclimate effects on plant community composition (Zellweger *et al*., 2020). In fact, there is evidence that the temperature triggering cell growth in the apical meristems of the buds is directly sensed within each individual bud, likely to be by the meristems themselves, as experimentally shown for species used in horticulture such as *Cucumis sativus* L. (Savvides *et al*., 2016). Similarly, daylength is perceived at the individual bud level by phytochromes within the leaf primordia (Zohner & Renner, 2015). When plants are growing in an open area, meristem temperature is generally higher compared with standard air temperature during the day and lower during the night, especially during bright days and clear nights due to shortwave and longwave radiative forcing and cooling, respectively (Savvides *et al*., 2013). More accurate microclimatic records reflecting the actual meristem temperature are therefore necessary to improve models of plant phenology and the associated physiological processes.

The timing of autumn leaf senescence is strongly regulated by autumn temperature and photoperiod in many temperate tree species (Keskitalo *et al*., 2005; Vitasse *et al*., 2009; Fu *et al*., 2018). However, spring and summer photosynthesis (Zani *et al*., 2020), CO_2_ concentration (Sigurdsson, 2001), soil nutrient status (Weih & Karlsson, 1999; Sigurdsson, 2001; Estiarte & Peñuelas, 2015; Fu *et al*., 2019a, 2019b,2019a, 2019b) and water availability (Xie *et al*., 2015; Arend *et al*., 2016a, 2016b,2016a, 2016b) can have a large influence as well. These factors have often been studied *in situ*, not accounting for micro‐environmental heterogeneities, but they have rarely been studied under experimental conditions (but see e.g. Arend *et al*., 2016b; Fu *et al*., 2019a, 2019b,2019a, 2019b; Zani *et al*., 2020), complicating conclusive results about their respective effects and interactions. In addition, inconsistent results have been found for the progress of leaf senescence in response to moderate drought, hot spells and nutritional status under natural conditions in different species (Estiarte & Peñuelas, 2015; Xie *et al*., 2018; Chen *et al*., 2020; Mariën *et al*., 2021), underscoring the importance of controlled experiments (but see Fu *et al*., 2019a, 2019b,2019a, 2019b; Zani *et al*., 2020).

Recently, leaf senescence timing of temperate trees has been proposed to be regulated by sink limitation of photosynthesis, which has been experimentally demonstrated under contrasting light conditions, temperature and CO_2_ levels (Zani *et al*., 2020). The hypothesis that photosynthesis is regulated by the strength of the carbon sink (i.e. the use of photoassimilates for growth) has been first formulated by Boussingault (1868). Accordingly, at the end of the season when tree primary and secondary growth ceases, there is an increasing imbalance between the production of carbohydrates (source) and their use for growth (sink). During this period, carbohydrates generally accumulate faster in leaves and other organs, even though they can be, to some extent, actively regulated by the plant (Dietze *et al*., 2014; Gilson *et al*., 2014). This excess of carbohydrates at a time when growth demand is limited could lead to a downregulation of the photosynthetic genes and accelerate the induction of leaf senescence (Paul & Foyer, 2001). In addition, environmental stress, such as limited water, high solar radiation or extreme temperature, has been shown to accelerate leaf senescence of temperate trees (Gallé *et al*., 2007). By interacting with endogenous factors (e.g. hormones), these environmental stressors can induce degradation of chlorophyll and photosystems, leading to a decline in the capacity to dissipate excess excitation energy in chloroplasts and, in turn, the accumulation of reactive oxygen species (ROS) and the acceleration of leaf senescence (Juvany *et al*., 2013). ROS concentration increases during drought‐induced leaf senescence (Munné‐Bosch & Alegre, 2004), but the ability to recover after such stress depends on the species and can be high as for example in pubescent oak (Gallé *et al*., 2007). As such, the sensitivity of leaf senescence to environmental stress appears to depend on species’ resistance strategies and the severity of the stressor, which can lead to contrasting results among co‐existing trees (e.g. delay rather than an advance of leaf senescence under moderate stress, see Xie *et al*., 2018). The regulation of leaf senescence therefore appears to result from a complex balance between sink and source strength and stress responses, which needs to be explored under controlled conditions to understand and forecast phenological changes under continued global warming.

Here we experimentally assessed the effects of light (‘sun’, 100% of photosynthetically active radiation (PAR) vs ‘shade’, *c*. 30% PAR) and bud albedo (white vs black‐painted buds) on budburst timing and the effect of light, soil water availability (irrigated, control and reduced precipitation) and soil nutrients (low vs high) on leaf senescence timing of 2–4‐yr‐old temperate trees (*Fagus sylvatica* L., *Fraxinus excelsior* L., *Prunus avium* L. and *Quercus robur* L.). We aimed to address the following questions:

1. To what extent is leaf‐out regulated by microclimatic conditions, that is does high bud albedo and shade delay leaf‐out at the individual level?
2. Do source–sink feedbacks and/or stress responses explain the effects of nutrient availability, solar radiation and soil moisture on autumn leaf senescence? Specifically, does elevated sink strength (high nutrients) lead to delayed senescence, does increased light availability (elevated photosynthesis) advance senescence, and how does water availability interact with these patterns?
3. Are the different responses among species related to their tolerance to drought or shade?

Assuming that bud meristems are the temperature‐sensitive part (‘thermometer’) of the plant, we expected that white‐painted buds and shade would delay budburst as a result of lower temperature experienced by buds. We expected earlier senescence under full sun conditions because carbohydrate reserves would be faster accumulated according to the sink‐limitation hypothesis, and/or due to higher oxidative stress damaging the photosystems (photo‐oxidative stress hypothesis), especially for shade intolerant species. Furthermore, we expected delayed leaf senescence under elevated nutrient availability as a result of increased sink strength, which may compensate the cost of maintaining leaves alive (Paul & Foyer, 2001). Finally, we expected a mixed response of the timing of leaf senescence to drought depending on species‐specific sensitive to drought.

## Materials and Methods

### Study species

We investigated microclimate and nutrient effects on leaf phenology of four species: *Prunus avium* L., *Fraxinus excelsior* L., *Fagus sylvatica* L. and *Quercus robur* L. For clarity and brevity, we refer from this point forwards to each species by its common name, that is cherry, ash, beech and oak, respectively. These species were selected due to their large variation in spring and autumn phenology and their differences in shade and drought tolerance. In the study area at the juvenile life‐stage, cherry and ash are amongst the first tree species to flush in spring and senesce in autumn, whereas beech and oak are rather late‐flushing and late‐senescing species (Vitasse *et al*., 2013 and see Supporting Information Fig. S1). Beech is the most shade‐tolerant species followed by cherry, ash and oak, whereas oak and cherry are more drought tolerant than ash and beech (see shade and drought tolerance indexes extracted from Niinemets & Valladares, 2006 in Table S1). Seedlings of each species except ash were purchased at a local nursery (Wiler, 455 m asl, 47°09′N, 7°33′°E) and came from local forests (see details in Table S1). The ash seedlings were taken from a forest near the experimental site (Lenzburg, 400 m asl, 47°24′N, 8°09′E) and were directly transplanted into the experimental boxes on 15 November 2018. Seedlings were 2‐ to 4‐yr‐old and *c*. 47 cm tall (see Table S1 for more details).

### Experimental design and treatments

The experiment took place in a common garden at WSL Research Institute in north‐eastern Switzerland (47°21′38″N, 8°27′16″E; 550 m asl; mean annual temperature 9.3°C, mean annual precipitation 1134 mm, MeteoSwiss station Fluntern, 1981–2010). The design consisted of 54 wooden containers (1 m × 1 m and 0.5 m deep) arranged in groups of three, which was the unit for climate manipulation (called from this point forwards ‘plot’; see Fig. S1). The 18 plots, containing each three containers, were then arranged in three rows (six plots per row), considered as blocks in the experimental design, to account for possible microclimatic heterogeneity, that is each treatment was replicated three times. Only the two outer containers were used per plot, which are from this point forwards referred to as mesocosms (*n* = 36). The central container was filled with soil but left without any plants (see Fig. S1). Each mesocosm was filled with a mixture of quartz sand, fibric peat, expanded schist and pumice, and the bottom of each mesocosm was covered by a permeable plastic foil to avoid water retention and ensure a good drainage after rainfall. This mixture was designed to be nutrient poor and sandy to facilitate soil nutrient and moisture manipulation by adding fertiliser and water, respectively (Zhang *et al*., 2020). On 15 November 2018, 20 seedlings were planted in each study mesocosm (four rows of five individuals), mixing and alternating two species per mesocosm. To ensure homogenous plant height and minimise competition for light, ash and cherry, and oak and beech respectively were planted together (cherry and ash were slightly taller than oak and beech, see Table S1). In total, 720 seedlings (4 species × 10 replicates × 6 treatments × 3 blocks) were planted and monitored for phenology and growth. Six treatments were used to analyse spring and autumn phenology, of which four treatments were used to test their effect on both spring and autumn phenology (Table 1). In the ‘sun treatment’, trees were exposed to full sun (100% PAR). In the ‘shade treatment’, trees were exposed to shade conditions, using a shading net that intercepted *c*. 70.3 ± 2.1% PAR (mean ± SE, PARmesocosm/PARambient × 100; measured on four different days in February and September 2019 between 13:30 and 15:30 under either sunny or cloudy conditions in all three blocks using a Li‐Cor Li189 quantum PAR light sensor). In the ‘drought’ treatment, natural rainfall was intercepted, using a roof with plastic channels that removed *c*. 50% of the ambient precipitation (using V‐shaped plastic channels mounted upwards at *c*. 2.5 m from ground above the plants and covering *c*. 50% of the mesocosm surface; see picture in Fig. S1). The ‘control‐drought’ treatment served as control for the drought treatment, using the same roof infrastructure as used in the drought treatment, but that allowed almost 100% precipitation throughfall (using V‐shaped plastic channels mounted downwards). Because the soil moisture between the drought and the drought‐control treatment differed significantly during the summer but not before budburst in which it remained relatively high (80–100% of the field capacity; Fig. S2a), these two treatments were only used to study the effects on autumn senescence. As additional budburst treatments, we modified the albedo of the buds by painting half of the buds of the plants either in black (low albedo treatment, called from this point forwards ‘black’ treatment) or in white (high albedo treatment, called from this point forwards ‘white’ treatment), using tinting dispersion paints (Schöner Wohnen Vollton‐ & Abtönfarbe*)* applied on 23 January 2019 (see photographs in Fig. S1). No potential deleterious impact of the paint was detected as the leaves emerged normally and were growing as much as the ones that originated from the nonpainted buds. According to the manufacturer, the painting does not contain relevant persistent, bioaccumulative and toxic substances. Plants with buds painted in white or black were kept in the same mesocosm, which reduced the replicates to 5 instead of 10 per block compared with the other treatments. After leaf‐out, the shade, sun, drought and control‐drought treatments were maintained through the growing season. To test the effect of nutrient and water availability on leaf senescence, we added two additional treatments after leaf‐out. In the ‘water’ treatment, the mesocosms were watered regularly, at least every week from 6 June to 24 October 2019 (25 times). Each mesocosm of the ‘high‐moisture’ treatment was watered manually for 2 min (two times for 1 min with a 5 min break in‐between) using a spray lance, which emitted 30 l water min^−1^. During a heatwave in June 2019, all mesocosms were watered manually for 5 s (6, 24 and 28 June 2019) to prevent mortality. In the ‘nutrient’ treatment, we added a substantial amount of slow‐release fertiliser (30 g of Gesal Floranid slow‐release lawn fertiliser in a granule form (composition 20% N, 5% P_2_O_5_, 8% K_2_O)) on 24 May and 29 July, by spreading the granules evenly on the surface of the mesocosms. Because the soil was extremely poor in nutrients, we added 5 g of this fertiliser to all the other mesocosms on 24 May 2019. These two treatments were assigned randomly to mesocosms that previously contained the black and white treatment plants (same conditions as the full sun treatment). Table 1 summarises the different treatments used during the spring and autumn phenology monitoring in 2019.

**Table 1:** Summary of the different treatments applied and their starting date to test their effect on the timing of budburst and/or leaf senescence.

Treatments	Treatment period	Tested impact on	
		Budburst	Leaf senescence
Full sun	Since 15 November 2018		
Full sun + low bud albedo (black‐painted buds)	23 January 2019 to leaf‐out		
Full sun + high bud albedo (white‐painted buds)	23 January 2019 to leaf‐out		
Full sun + fertilisation	24 May 2019 and 29 July 2019		
Full sun + irrigation	6 June 2019 to 24 October 2019 (25 times)		
Shade (shading net intercepting 70% of incoming radiation)	Since 24 January 2019		
Drought (roof intercepting 50% of the rainfall)	Since 24 January 2019	^a^	
Control drought (roof with rain shelter turned upside down)	Since 24 January 2019	^a^	

^a^ The effect of drought vs control drought was not analysed for budburst timing as no significant difference in the soil moisture was observed between the two treatments before budburst.

### Microclimatic measurements

Soil moisture was recorded in every mesocosm at 30 min intervals using EC‐5 soil moisture sensors (Decagon, Pullman, WA, USA) measuring volumetric soil water content. Because these sensors are rather sensitive to differences in soil compaction, we standardised the records of each sensor by the value obtained after irrigating the mesocosm at saturation on 21 November 2019 (using the mean value between 06:00 h and 10:00 h on the following day, that is *c*. 14 h after the irrigation). Therefore, soil moisture is given as % of full saturation (field capacity), which accounts for absolute deviation among the sensors and provides a standardised comparison among the treatments.

Air temperature was recorded in each plot every hour using EL‐USB‐2+ sensors (Lascar Electronics, Salisbury, UK) covered by a radiation shield (TFA Dostmann GmbH, Wertheim, Germany) from 25 January 2019 until December 2019. Additionally, air temperature at a height of 2 m was also recorded outside of the plots under an aluminium radiation shield every 30 min. The second half of February 2019 was particularly warm, with daily maximum temperatures consistently being above the long‐term average (Fig. S3). The last frost days with temperature down to −1.5°C occurred on 5–7 May (day of the year (DOY) 125–127), when all species had already leafed out (Fig. S3), but only slight frost damage was observed on beech seedlings. Two marked warm spells occurred at the end of June to beginning of July with daily maximum temperature higher than 35°C during a consecutive 7 d (DOY 180–186; Fig. S3) and at the end of July (DOY 209–211; Fig. S3). No frost occurred in autumn before leaf senescence reached 50% for any of the species (first autumnal frost on 13 November, DOY 317; Fig. S3).

**Fig. 1:**
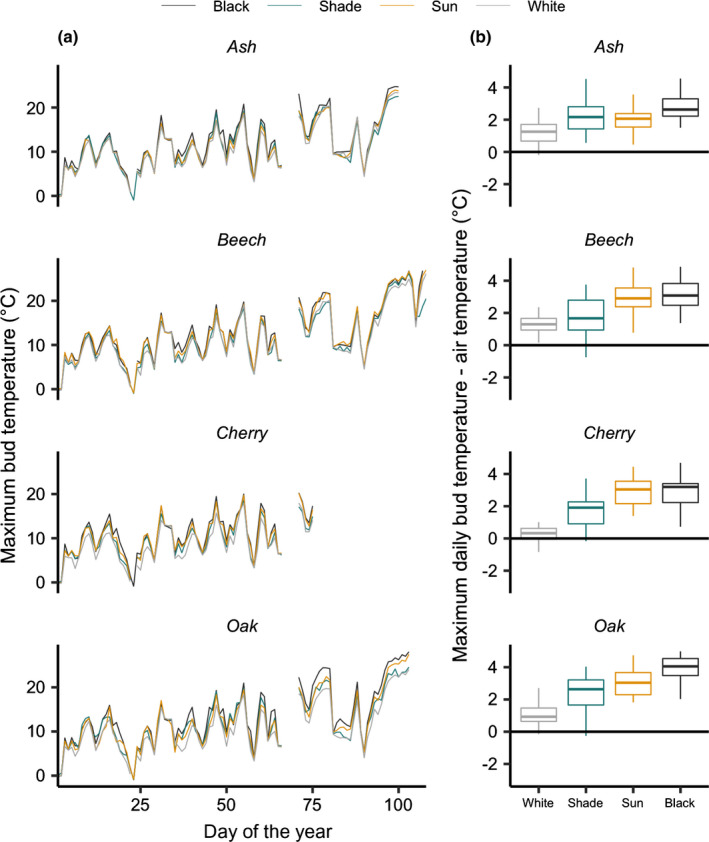
Daily maximum temperatures recorded within the bud from January 2020 until species‐specific budburst. (a) Daily maximum temperatures recorded within the buds for the different species and treatments. (b) Boxplots (first quartile, median and third quartile ± minimum and maximum values within 1.5 times the interquartile range) of the difference between daily maximum bud and air temperature on days with solar radiation reaching up at least 400 W m^−2^ from 26 January to budburst between 12 and 16 h. This includes 26, 48, 53 and 58 d for cherry, ash, oak and beech, respectively.

Bud temperature was recorded in the following year (2020) from 1 January until species‐specific budburst by inserting the needle (0.3 mm diameter and 13 mm long) of a thermocouple probe inside buds (Thermocouple Probe Model HYP1 ©OMEGA, Omega Engineering Inc., Norwalk, CT, USA; Fig. S1). Bud temperature was recorded for two individuals from different blocks for each of the four species and for each of the following treatments: black‐painted buds, white‐painted buds, shade, full sun. All the 32 thermocouples (4 species × 2 replicates × 4 treatments) were connected to a datalogger that recorded bud temperature every 10 min. Some thermocouple probes were disconnected from the buds due to a storm that occurred on 6 March 2020 and were inserted again in the respective buds 4 d later on 10 March. We discarded all records between these two dates. We averaged the temperature of the two replicates for each species and treatment and computed the daily minimum, mean and maximum values. Additionally, air temperature at plant canopy height was recorded every 10 min with the same thermocouple probes protected from direct solar radiation with a custom‐fabricated radiation shield with several layers of carton recovered by aluminium foils (see details in Frei *et al*., 2020). This latter measurement was used as a reference to compare bud and air temperature in Fig. 1(b).

### Phenology monitoring, growth measurements and soil inorganic nitrogen

Bud development and leaf senescence were monitored for all 720 individual seedlings in spring and autumn 2019. Bud development in spring was monitored by the same observer weekly or twice a week during warmer periods, from 15 February until 24 May, that is when the last individual unfolded its leaves. Bud development was monitored using a four‐stage categorical scale (Vitasse, 2013): stage 0 (dormant bud), no bud development visible; stage 1 (bud swelling), buds swollen and/or elongating; stage 2 (budburst), bud scales open and leaves partially visible; stage 3 (leaf‐out), leaves fully emerged from the buds but still folded, crinkled or pendant, depending on species; stage 4 (leaf unfolded), at least one leaf fully unfolded. For each tree, the day of year when the first bud reached the respective stage was recorded. The stages were estimated by linear interpolation when necessary (i.e. when a given stage occurred in between two monitoring dates).

For leaf senescence in autumn, we evaluated the percentage of coloured or fallen leaves for every seedling according to the method developed in Vitasse *et al*. (2009) on a weekly basis from 23 August to 29 November. As a proxy for the beginning, middle and end of the leaf senescence process, for each individual tree, we computed the date when 25%, 50% and 75% of leaves were either coloured or had fallen using linear interpolation between two monitoring dates.

Measurements of seedling height and diameter were conducted at 2 cm above plant collar on all individuals before budburst and after leaf fall in spring and autumn 2019, respectively, using a graduated pole and an electronic caliper. We estimated the above‐ground biomass using the allometric equation provided by Annighöfer *et al*. (2016) as follows:

$$\text{AGB}=\beta_{1}\times {\left({\text{RCD}}^{2}\times H\right)}^{\beta_{2}}$$

with AGB = above‐ground biomass (g); RCD = root collar diameter (cm); *H* = height (cm); and b1 and β_1_ and β_2_ = species‐specific coefficients as provided in Table 4 from Annighöfer *et al*. (2016).

We computed the biomass increment during the growing season 2019 by subtracting the AGB estimated in spring 2019 from the AGB estimated in autumn 2019. These biomass increment measurements were used to characterise plant responses to the different treatments and to interpret the leaf senescence observations.

We measured extractable inorganic N by sampling soil in each mesocosm using a soil corer at 10 cm depth (missing three samples for each mesocosm on 3 September 2019). We used ion exchange by adding KCl solution to extract nitrate and ammonium from the soil (Table S2).

### Data analysis and statistics

The progress of bud development in spring and leaf coloration in autumn was modelled by using generalised additive mixed models (GAMMs) with a binomial distribution using the R package gamm4 v.0.2‐5. Bud development stages (0–4) in spring were transformed to fit a 0–1 range by dividing each stage by 4 to apply the binomial distribution. The values of the stages were then back transformed for the visualisation of the graph. For each species, the models included a smoothing spline with four degrees of freedom for the DOY with the treatment as a factor modulating the spline and the block as a grouping variable for the random intercept with individuals nested inside the block. The fitted GAMMs with the associated means and confidence intervals are shown. The GAMMs were used to get the overall time course of bud development in spring and leaf senescence in autumn depending on the treatments. We assessed the effect of the treatments on the time of budburst and leaf senescence across species and within species with linear mixed effect models using the lme function of the R package nlme v.3.1‐149 focusing on the DOY corresponding to stage 2 in spring (budburst) and to 50% senescence in autumn. We used block as random effect and treatments as fixed effects. Estimated marginal means were extracted from the model with the associated 95% confidence intervals. Post‐hoc tests using Tukey’s honest significant difference (HSD) tests were performed to test for significant differences between the control plants and the corresponding treatment. Analyses of stage 3 and stage 4 of spring bud development and of 25% and 75% autumn senescence yielded similar results and are therefore not shown. All data analyses and statistics were performed using R v.4.0.2 (R Core Team, 2020).

## Results

### Treatment effects on annual growth increment

Additional irrigation significantly increased growth for oak (+43%) and only marginally for ash (+39%; Table 2). Biomass increment was lower under shade conditions compared with the control for all species, but this was significant for cherry only (−37%) and marginally significant for beech (−19%; Table 2). No significant effect was found for the drought treatment for any species (Table 2), suggesting that the reduced soil moisture was not impairing growth further than in the control drought. In none of the species did the nutrient‐addition treatment lead to significantly increased growth compared with the control (Table 2).

**Table 2:** Estimated above‐ground biomass increment in 2019 (g dry weight, mean ± 1SE) for each treatment and species.

	Cherry	Ash	Oak	Beech
Control (full sun)	9.02 ± 0.92	1.21 ± 0.16	5.66 ± 0.51	7.94 ± 0.71
Shade	5.72 ± 0.4**	1.03 ± 0.13	4.74 ± 0.45	6.44 ± 0.52*
Water	9.08 ± 1.00	1.68 ± 0.18*	8.07 ± 0.60**	8.73 ± 0.75
Nutrient	9.85 ± 1.09	1.23 ± 0.19	6.63 ± 0.65	7.78 ± 0.81
Control drought	8.95 ± 0.83	0.93 ± 0.13	4.88 ± 0.41	7.77 ± 0.53
Drought	7.86 ± 0.75	0.86 ± 0.13	5.00 ± 0.35	7.52 ± 0.50

The biomass increment was computed using an allometric equation based on height and diameter with species‐specific parameters provided by Annighöfer *et al*. (2016) (see details in the Materials and Methods section). Asterisks indicate significant differences between a given treatment and its corresponding control (i.e. *full sun* for the treatment *shade*, *water* and *nutrient* and *control drought* for the treatment *drought*), tested with a mixed effect ANOVA with block as random effect and treatment as a fixed effect: **, *P* < 0.01; *, *P* < 0.1.

### Effect of bud albedo and light intensity on bud temperature

Bud temperature recorded in 2020 from January to budburst showed similar minimum temperature between the shade, full sun and white or black‐painted buds, irrespective of species (the mean difference ranged within 0.4°C for the different species from 1 January to DOY 75; Fig. S4). However, the daily maximum temperature was warmer in black‐painted buds and in buds fully exposed to sun than in white or shaded buds (Fig. 1a), especially during bright days. When selecting the days when solar radiation reached at least 400 W m^−2^ from 1 January until the budburst of each species (from 26 to 58 d depending on the species), daily maximum temperature recorded in black‐painted buds was on average 3.1°C (ash), 3.2°C (beech), 3.3°C (cherry) and 4.6°C (oak) warmer than air temperature, whereas white‐painted buds were only 0.2–1.3°C warmer than air temperature (Fig. 1b). Bud temperature measured in the shade was slightly warmer than the temperature of the white‐painted buds and generally cooler than buds fully exposed to sun (Fig. 1).

### Effect of bud albedo and light intensity on bud development

**Fig. 2:**
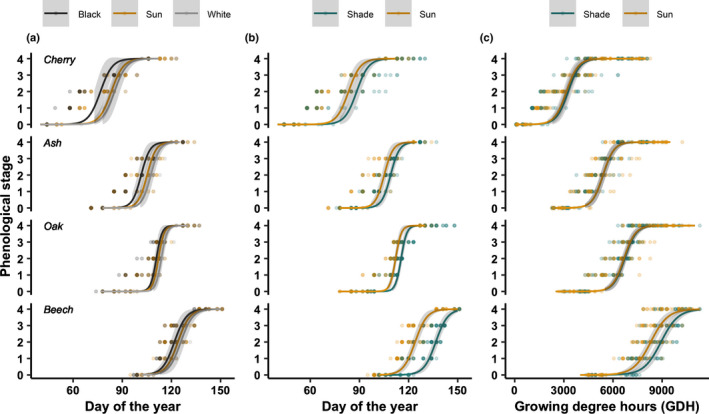
Bud development progress from closed buds (stage 0) to unfolded leaves (stage 4) in spring 2019 for seedlings with black‐painted and white‐painted buds compared with control seedlings (a), and for seedlings under shade condition compared with the control (sun) using either day of the year (b) or growing degree hours recorded within the plot (c) as *x*‐axis. Lines represent predictions from GAMMs using block as a random factor and a binomial distribution; 95% confidence intervals are represented in shaded areas.

Spring phenology significantly varied among species (Table 3), with cherry generally being the first species to leaf‐out (budburst DOY 75.7 ± 1.1; mean ± SE), followed by ash (DOY 102.8 ± 5.7), oak (DOY 111.6 ± 3.2) and beech (DOY 125.3 ± 7.4), irrespective of the treatment (Figs 2a, S3).

**Table 3:** Summary of the fixed‐terms of the mixed effect models testing the effects of species, treatments and their interactions on the budburst and leaf senescence dates (DOY), while accounting for the block effect as random effect.

Treatments	Budburst			Leaf senescence		
	df	*F*	*P*	df	*F*	*P*
Bud albedo (white/black/control)						
Species	3	899.0	< 0.0001		–	–
Treatments	2	24.8	< 0.0001		–	–
Species × Treatments	6	2.0	0.063		–	–
Shade (shade/sun)						
Species	3	656.9	< 0.0001	3	103.1	< 0.0001
Treatments	1	55.9	< 0.0001	1	221.2	< 0.0001
Species × Treatments	3	5.6	0.001	3	48.8	< 0.0001
Nutrients (nutrient/control)						
Species	–	–	–	3	400.8	< 0.0001
Treatments	–	–	–	1	5.9	0.016
Species × Treatments	–	–	–	3	0.97	0.410
Water (water/control)						
Species	–	–	–	3	331.3	< 0.0001
Treatments	–	–	–	1	15.87	0.0001
Species × Treatments	–	–	–	3	18.07	< 0.0001
Drought effect (drought/control drought)						
Species	–	–	–	3	198.3	< 0.0001
Treatments	–	–	–	1	< 0.1	0.925
Species × Treatments	–	–	–	3	3.8	0.011

Budburst, stage 2; leaf senescence, 50% of leaves coloured or fallen.

**Fig. 3:**
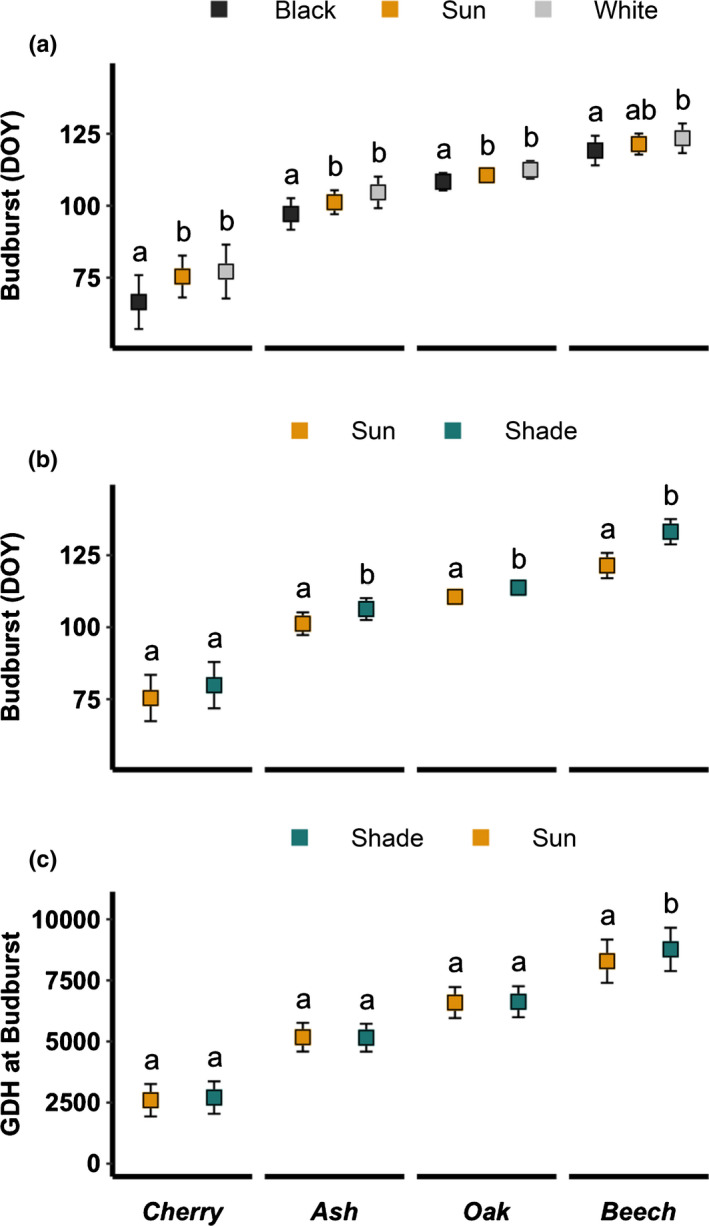
Budburst date (stage 2, day of the year (DOY)) (a, b) and growing degree hours (GDH) at budburst (c). Values correspond to the marginal mean estimates of the mixed effect ANOVA with blocks as random factor. The error bars correspond to the confidence intervals at 95%. The models were performed separately for each species and pairs of treatments shown in each panel. For each species, different letters among treatments indicate significant differences (post‐hoc Tukey’s tests at α = 0.05).

Bud albedo (white‐painted vs black‐painted buds) consistently affected the time of budburst across all species (Table 3). Seedlings with black‐painted buds started bud development significantly earlier than seedlings with white‐painted buds (Fig. 2a), especially for early flushing species (budburst cherry: −10.6 d; ash: −7.5 d; oak: −4.1 d; and beech: −4.3 d; Fig. 3a). Seedlings with no painting buds started bud development later than seedlings with black‐painted buds and slightly earlier than seedlings with white‐painted buds (Figs 2a, 3a).

Lower solar radiation significantly affected spring bud development, with later bud development under shaded conditions for all species (Table 3; Fig. 2b). Specifically, budburst was delayed by 4.5 d for cherry (not significant), 5.1 d for ash, 3.2 d for oak and 11.8 d for beech (Fig. 3b). These delays could be explained by lower temperatures in the shade treatments compared with the controls (Figs 3c, S5). Indeed, when accounting for this difference by accumulating GDH (growing degree hours accumulated above 5°C), all species required a similar amount of GDH until budburst (Figs 3c, S5a). Only the late‐leafing beech required 483 additional GDH to budburst under shade conditions (Figs 3c, S5a).

### Effect of nutrients and irrigation on leaf senescence

**Fig. 4:**
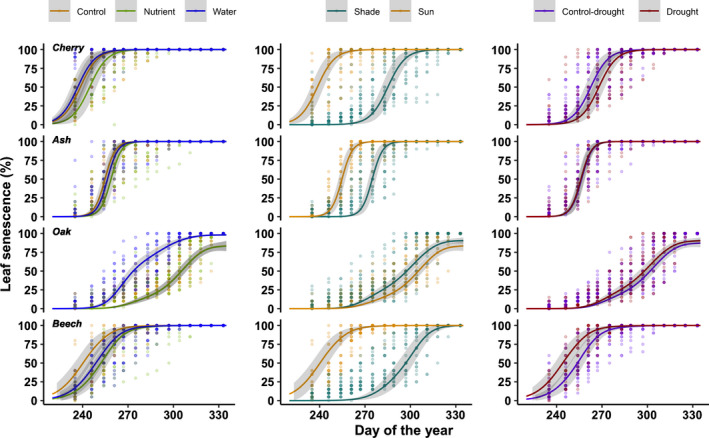
Progress of leaf senescence in autumn 2019 within the different treatments. Lines represent the predictions from GAMMs using block as a random factor and a binomial distribution; 95% confidence intervals are represented in shaded areas.

**Fig. 5:**
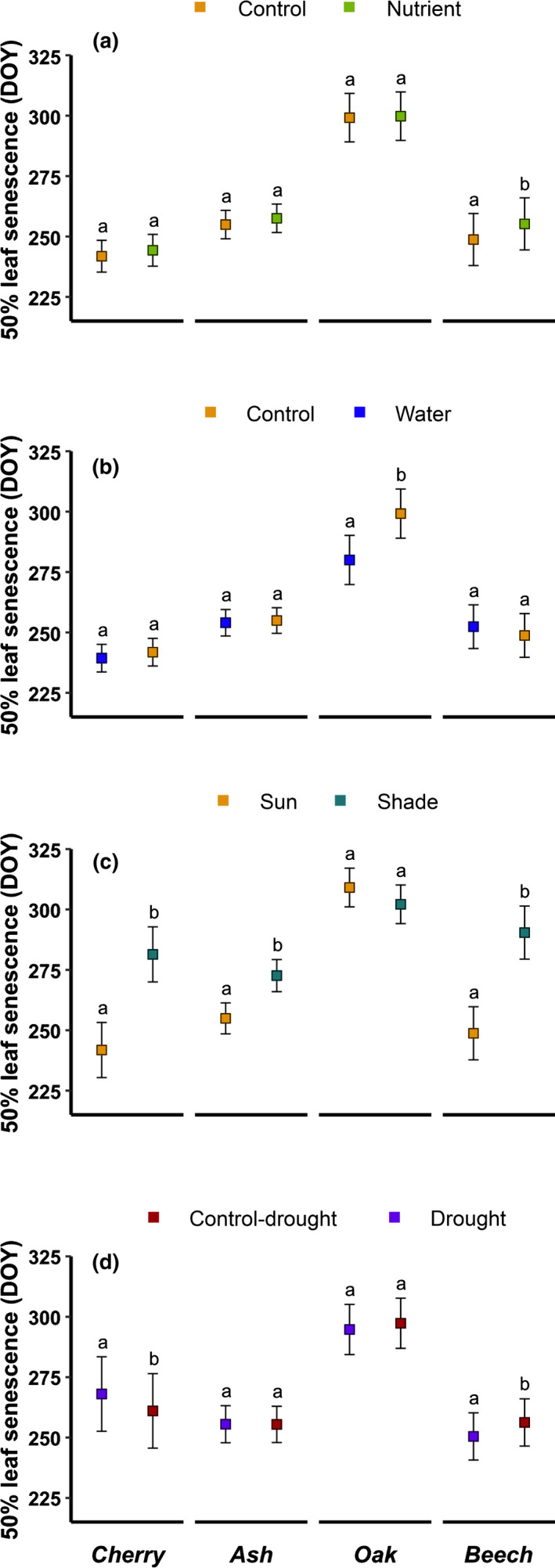
Date of leaf senescence at 50% rate estimated from the mixed effect models using block as random effect for the different treatments (a, nutrient; b, irrigation; c, shade; d, drought) in comparison with their corresponding control. Values correspond to the marginal mean estimates of the mixed effect ANOVA with blocks as random factor. The error bars correspond to the confidence intervals at 95%. The models were performed separately for each species and pairs of treatments shown in each panel. For each species, different letters among treatments indicate significant differences (post‐hoc Tukey’s tests at α = 0.05).

Irrigation and nutrients had a significant effect on leaf senescence, but the effect differed among species as shown by the significant interaction between species and treatments (Table 3). Only in oak did irrigation have a significant effect on leaf senescence (Fig. 4a), with an advance of 19.8 d compared with the control based on the 50% leaf senescence stage (Fig. 5b). Except for oak, nutrients tended to delay the date of 50% leaf senescence compared with the control treatment (Fig. 4a), but this delay was significant for beech only (+6.5 d).

### Effect of light intensity on leaf senescence

Leaf senescence was strongly delayed under shaded conditions for all species except oak in which a slight insignificant advance was found (Table 3; Figs 4b, 5c). Shade conditions delayed senescence by +39.6 d, +17.7 d and +44.8 d for cherry, ash and beech, respectively, whereas air temperature measured under a radiation shield was slightly cooler in the shade treatment (Fig. S6). For oak, 50% leaf senescence occurred 5.5 d earlier under shade conditions (not significant; Fig. 4c) and 75% leaf senescence occurred 7.0 d earlier (*P* = 0.011).

### Effect of reduced precipitation on leaf senescence

Overall, the drought treatment had no significant effect on leaf senescence across species (Table 2). However, the species‐specific analysis showed that lower soil moisture during summer significantly delayed leaf senescence of cherry by +7.0 d and significantly advanced senescence of beech by −7.1 d (Figs 4c, 5d). No effect was found for the two other species (Figs 4c, 5d).

## Discussion

Our experimental study shows that the microclimate in which seedlings are growing significantly affects leaf phenology both in spring and in autumn. The albedo treatment, in which we painted buds to modify heat reflectance, demonstrates that temperature is sensed at the bud level: black‐painted buds with lower albedo and higher maximum bud temperature during bright days showed earlier bud development relative to white‐painted and unpainted buds. Moreover, a cooler microclimate induced by the shading nets significantly delayed budburst timing of all species. This delay was mainly explained by growing‐degree‐day accumulation, whereby the shaded and control plants required similar warming sums until budburst.

Regarding autumn phenology, reduced light intensity led to reduced biomass increment in all species (but significant for cherry and beech only) and strongly delayed leaf senescence for all species but oak. The magnitude of such delay exceeds the interannual variability observed over several decades in Switzerland for common tree species or for beech in France (Delpierre *et al*., 2009; Bigler & Vitasse, 2021; Meier *et al*., 2021). This suggests that the phenological cycles of understory trees are strongly affected by the shade imposed by overstory trees and that trees growing under low light compensate for the reduced photosynthetic assimilation by extending their growing season. In addition, our results showed that higher water availability can strongly advance leaf senescence when it significantly increases growth rate (as found for oak). Overall, nutrients and water availability had only little effect on growth that might also explain their limited impact on leaf senescence timing.

### Bud temperature as the main driver of budburst timing

Our study showed that black‐painted buds started their development earlier than nonpainted or white‐painted buds, suggesting that bud albedo affects internal physiological processes by influencing the temperature of bud tissues. Temperature records within buds show that black‐painted buds are up to 3.6°C warmer than white‐painted buds during bright days (mean difference for oak when solar radiation was more than 400 W m^−2^). The discrepancy in the time of budburst between black and white buds was more pronounced for early (cherry, ash) than for late‐flushing species (oak, beech). Because of the nonlinear response of bud development to spring warming temperatures, lower bud albedo early in spring may lead to a strongly increased accumulation of temperature relevant for bud cell growth, whereas this increase in effective heat sums might be less pronounced later in spring when days are already warm. In addition, early flushing species might be more sensitive to temperature increases than late‐flushing species for which more pronounced photoperiod and/or chilling requirements may limit responsiveness to spring warming (Fu *et al*., 2019a, 2019b,2019a, 2019b; Montgomery *et al*., 2020). Our results further demonstrate that lower radiation induced by the shading net substantially changed the microclimate of the buds, delaying bud development of beech by 12 d, which roughly correspond to half of the interannual variability that can be observed over several decades (Meier *et al*., 2021). The slower accumulation of growing degree days under shaded conditions fully explained the discrepancy in budburst timing between shaded and control plots, for all species except beech, which overall confirms that growing degree days/hours is a good method to predict budburst of temperate trees in regions where chilling is not limiting (Vitasse *et al*., 2018). However, the remaining discrepancy found for beech suggests that, in addition to temperature and photoperiod (Vitasse & Basler, 2013), light intensity may play a direct role in the regulation of bud development of European beech, as also suggested in a previous study analysing the partial correlation of leaf‐out timing of European trees and insolation (Fu *et al*., 2015a, 2015b,2015a, 2015b). Alternatively, this species may have a different threshold above which temperature is accumulated or may respond nonlinearly to forcing temperature. Further experiments controlling both light intensity and bud temperature are needed to clarify the potential effect of solar radiation on European beech phenology.

Bud temperature differs from air temperature measured by standard weather stations, that is under ventilated and shaded conditions. Under clear sky conditions, buds are heating up during the day by shortwave radiation and cooling down during the night by losses of heat from the atmosphere to the outer space through longwave radiation (radiative cooling). We therefore suggest that standard weather stations may substantially underestimate bud temperature during the day, when solar radiation is high and overestimate minimum temperature during the night when the sky is clear. Our temperature data recorded inside buds clearly show this effect for daily maximum temperature. Because sky brightness has been shown to have substantially increased since the 1980s in Europe, especially in spring (Sanchez‐Lorenzo *et al*., 2015; Pfeifroth *et al*., 2018), the discrepancy between standard air temperature and bud temperature may have substantially increased over recent decades. This may introduce a significant bias in phenology modelling, for example in the estimation of spring phenological sensitivity to temperature, which has been suggested to have decreased since the 1980s (Fu *et al*., 2015a, 2015b,2015a, 2015b). Our study calls for more investigations on how bud temperature differs from standard air temperature, depending on other important climatic factors such as solar radiation or wind.

### Factors affecting leaf senescence

Leaf senescence is assumed to occur when the cost of maintaining active leaves outweighs the benefits of photosynthesis and is seen as a strategy for reabsorbing nutrients from the leaves and reallocating them throughout the plant (Kikuzawa, 1991; Estiarte & Peñuelas, 2015). It has long been thought that the leaf senescence process of temperate trees is mainly triggered by the decrease in temperature and photoperiod during autumn (Delpierre *et al*., 2009; Liu *et al*., 2020). However, recently, other factors have been suggested to influence leaf senescence, such as temperature during the growing season (Liu *et al*., 2019, 2018), water (Liu *et al*., 2019) and nutrient availability (Weih, 2009; Fu *et al*., 2019a, 2019b,2019a, 2019b), summer drought stress (Schuldt *et al*., 2020) and light conditions (Wingler *et al*., 2006). These factors can have a direct effect on leaf senescence by inducing a stress response or affect leaf senescence indirectly by modulating the plant source–sink relationship (Paul & Foyer, 2001), which plays a prominent role in the senescence process (Zani *et al*., 2020). The relative effects of these drivers often depend on the species. For example, severe drought has been shown to hasten leaf senescence of temperate trees at low elevations (Hwang *et al*., 2014; Xie *et al*., 2015), which might be a strategy to reduce transpiration (Munné‐Bosch & Alegre, 2004) and avoid xylem embolism (Bréda *et al*., 2006) or the direct consequence of vessel cavitation under extreme severe drought as observed for European beech in central Europe during the summer 2018 (Schuldt *et al*., 2020; Wohlgemuth *et al*., 2020). Our results also showed earlier senescence under drought compared with the control‐drought plots for beech and delayed senescence in the irrigated plots. By contrast, we found the opposite pattern for oak with significantly earlier senescence and elevated growth in the irrigated plots and slightly delayed senescence in the drought treatments. We expected drought to affect carbon sink and source strength and therefore senescence timing. However, the drought treatment led to significantly lower soil moisture content during late summer only, whereas the difference with the control was negligible at the beginning of the growing season until mid‐July, that is when most of the growth may have already occurred. Accordingly, no differences in the estimated biomass increment was found between the drought and control treatment after the growing season. It is widely recognised that severe droughts in spring strongly affect growth rate (Vitasse *et al*., 2019; Bose *et al*., 2021) and may therefore affect leaf senescence timing due to lesser carbon supply and growth. More investigations should be conducted to determine the seasonal effects of drought on leaf senescence. Opposite responses of leaf senescence to moderate drought stress were also found for temperate trees in the north‐eastern United States, with a delay in ash, maples and birches and an advance in oak and beech (Xie *et al*., 2018). The heterogeneity among microenvironments could partly explain this pattern when leaf senescence is studied *in situ*. Here, we rule out the possibility of different microenvironments and attribute these opposite responses to species‐specific physiological features/characteristics (e.g. tolerance to drought or shade). For instance beech is a shade‐tolerant species with rather low tolerance to drought, whereas pedunculate, sessile and pubescent oaks are more tolerant to drought (Gallé *et al*., 2007; Rubio‐Cuadrado *et al*., 2018; Vitasse *et al*., 2019), and capable of maintaining photosynthesis when leaf water potential is low (Raftoyannis & Radoglou, 2002). The species‐level variation in the responses to drought might be the result of differences in the relative importance of stress responses vs sink limitation. Reduced water availability can decrease photosynthetic activity, at least in drought‐intolerant species, which in turn delays the senescence process by delaying saturation of a trees’ annual carbon sink (Zani *et al*., 2020). Conversely, drought might have a direct effect on leaf senescence, whereby intense droughts cause a stress reaction, increasing ROS concentration in leaves or even hydraulic failure leading to precocious leaf senescence.

Increased nutrient availability has generally been shown to delay leaf senescence. For instance, high nutrient availability was found to delay leaf senescence in *Populus trichocarpa* in Iceland (Sigurdsson, 2001) and in seedlings of *Aesculus hippocastanum* and beech (Fu *et al*., 2019a, 2019b,2019a, 2019b). By contrast, under low nutrient availability, elevated CO_2_ concentration was found to accelerate growth cessation in *Populus trichocarpa* (Sigurdsson, 2001). Sigurdsson (2001) suggested that under elevated CO_2_ or under low nutrient availability, there is an imbalance between carbon and nitrogen sources which alters autumn phenology. Other studies have suggested that a potassium deficiency may lead to earlier leaf senescence (Wang *et al*., 2012; Pan *et al*., 2017), probably because, in addition to the negative impacts on photosynthesis, K deficiency hinders the export of sucrose from the leaves through the phloem (Cakmak, 2005). Senescence, therefore, appears to be largely driven by the interaction between nutrient, particularly nitrogen, and carbon supply (Paul & Foyer, 2001). High nutrients will therefore allow trees to maintain source activity (photosynthesis) for longer time and shed their leaves later in the year. However, no significant increase in biomass was found in our experiment for the fertilised treatments, which may explain the insignificant delays in leaf senescence observed for beech, cherry and ash in this treatment. It is possible that the additional fertiliser was not yet absorbed by the trees, or only partially, as suggested by the high nitrate concentration remaining in the soil at the end of the season (25 times higher in the fertiliser treatment than in the control for oak/beech and *c*. nine times higher for cherry/ash, see Table S2).

We found that reduced light availability (reduction of PAR by *c*. 70%) strongly delayed leaf senescence of ash, cherry and beech by 18, 39 and 42 d along with a reduction of biomass increment of 15%, 37% and 19%, respectively. This can also be explained by the sink‐limitation hypothesis (Wingler *et al*., 2006; Wingler & Roitsch, 2008; Dox *et al*., 2020). Shaded conditions over summer led to reduced carbon uptake (i.e. lower biomass increment) due to lower photosynthetic activity (Sevillano *et al*., 2016), and subsequently delayed the senescence process. This result was also found for common sunflower and beans (Ono *et al*., 2001) or for European beech and the Japanese spiraea (Zani *et al*., 2020). It remains an open question to which degree sink limitation operates at the leaf, branch or whole‐plant level. Given that the source/sink control of leaf senescence appears to be largely driven by leaf‐level nitrogen to carbon ratios (Paul & Foyer, 2001), localised effects on leaf senescence can be expected. This agrees with observations that, under natural conditions, the upper part of the canopy, which is more exposed to full light, shows earlier senescence than more shaded parts of the tree (Gressler *et al*., 2015).

In addition, photo‐oxidative stress might drive early senescence under high light. Photo‐oxidative stress occurs when light‐energy absorption exceeds the capacity for light utilisation: an excess of photons may lead to nonphotochemical quenching and oxidative stress by an accumulation of ROS (Müller *et al*., 2001). This may lead to photoinhibition (Long *et al*., 1994) and can accelerate the process of senescence (Munné‐Bosch & Alegre, 2004; Juvany *et al*., 2013; Pintó‐Marijuan & Munné‐Bosch, 2014). Photo‐oxidative stress might, therefore, play a role, especially in species adapted to grow under the canopy shade at juvenile age such as beech. The absence of a response to light or drought in oak could be related to its tolerance to high solar radiation (Valladares *et al*., 2002) and its ability to efficiently dissipate an excess of energy and degrade ROS under photo‐oxidative stress, as demonstrated for pubescent oak (Gallé *et al*., 2007).

Overall, in addition to the well known effects of temperature and photoperiod on the regulation of leaf senescence timing, our results suggest a tight interplay between source and sink processes in regulating the end of the seasonal vegetation cycle, which can be largely influenced by light, water and nutrient availability. More experiments will be necessary to fully untangle the relative contribution of direct effects of solar radiation on leaf senescence in relation to the indirect effects mediated through sugar and nutrient availability.

### Conclusion

This study demonstrates the importance of microclimatic conditions, especially solar radiation, in regulating the timing of budburst in spring and leaf senescence in autumn. While our experiment shows that light availability mainly affects spring budburst through modification of bud temperatures, in European beech, light intensity and/or quality may directly affect budburst as the delayed budburst under shaded conditions could not be fully explained by the local temperature recorded beneath the shading net. A potential avenue to improve phenological predictions will therefore be to quantify how bud and leaf temperatures differ from standard air temperature depending on other meteorological factors, such as solar radiation and wind. Light availability also had a large effect on autumn senescence, with a considerable delay of leaf senescence under shaded conditions during the growing season found for all species except oak along with a reduction of growth. This delay under low light can be explained by the sink‐limitation hypothesis, whereby leaf senescence is tightly linked to photosynthate and nutrient supply. Oxidative stress under high light conditions may further drive this trend in late successional and shade‐tolerant species sensitive to heat and drought, such as European beech. The results provide important insights into the roles of sink limitation and drought stress in mediating autumn phenology and call for a more accurate representation of microclimate to improve phenological predictions.

## Author contributions

YV, FB and BM planned and designed the experiment. YV conducted the experiment with the field assistance of RK and MGW. CMZ and YHF helped in the interpretation of the results. YV analysed the data and drafted the manuscript with substantial inputs from all co‐authors.

## Supporting information


**Fig. S1** Picture showing the experimental infrastructure used in the study.
**Fig. S2** Soil moisture of the different treatments during the experiment.
**Fig. S3** Air temperature at 2 m height during the experiment.
**Fig. S4** Daily minimum temperatures recorded within the bud from January 2020 until species‐specific budburst in white, black, shade or fully exposed buds.
**Fig. S5** Growing degree hours above 5°C from 1 February to budburst in the shade and sun treatment.
**Fig. S6** Air temperature recorded at canopy height during summer 2019 in the shade and sun treatment.
**Table S1** Seed material used in the experiment.
**Table S2** Amount of total nitrogen (N), nitrate (NO_3_^−^) and ammonium (NH_4_^+^) in the soil for each of the mesocosms at the end of the growing season (3 September 2019).
Please note: Wiley Blackwell are not responsible for the content or functionality of any Supporting Information supplied by the authors. Any queries (other than missing material) should be directed to the *New Phytologist* Central Office.

## Data Availability

The data that support the findings of this study are available from the corresponding author upon request.
