# Supplementary material for: Impact of microclimatic conditions and resource availability on spring and autumn phenology of temperate tree seedlings
Source: New Phytol. 2021 Aug 16;232(2):537–50. doi: 10.1111/nph.17606 (PMC8518844; doi:10.1111/nph.17606)
Supplement: Supplementary file 1 — Fig. S1 Picture showing the experimental infrastructure used in the study. Fig. S2 Soil moisture of the different treatments during the experiment. Fig. S3 Air temperature at 2 m height during the experiment. Fig. S4 Daily minimum temperatures recorded within the bud from January 2020 until species‐specific budburst in white, black, shade or fully exposed buds. Fig. S5 Growing degree hours above 5°C from 1 February to budburst in the shade and sun treatment. Fig. S6 Air temperature recorded at canopy height during summer 2019 in the shade and sun treatment. Table S1 Seed material used in the experiment. Table S2 Amount of total nitrogen (N), nitrate (NO3 −) and ammonium (NH4 +) in the soil for each of the mesocosms at the end of the growing season (3 September 2019). Please note: Wiley Blackwell are not responsible for the content or functionality of any Supporting Information supplied by the authors. Any queries (other than missing material) should be directed to the New Phytologist Central Office. [file NPH-232-537-s001.pdf]

***New Phytologist* Supporting Information**

Article title: Impact of microclimatic conditions and resource availability on spring and autumn phenology of temperate tree seedlings

Authors: Yann Vitasse, Frederik Baumgarten, Constantin Zohner, Rungnapa Kaewthongrach, Yongshuo H. Fu, Manuel Walde, Barbara Moser

Article acceptance date: 02 July 2021

The following Supporting Information is available for this article (short titles):

Table S1. Seed material used in the experiment.

Table S2. Amount of total nitrogen (N), Nitrate (NO_3^-) and ammonium (NH_4^+) in the soil for each of the mesocosms at the end of the growing season (3 September 2019).

Fig. S1. Picture showing the experimental infrastructure used in the study.

Fig. S3. Soil moisture of the different treatments during the experiment.

Fig. S3. Air temperature at 2m height during the experiment.

Fig. S4. Daily minimum temperatures recorded within the bud from January 2020 until species-specific budburst in white, black, shade or fully exposed buds.

Fig. S5. Growing degree hours above 5°C from 1 February to budburst in the shade and sun treatment.

Fig. S6. Air temperature recorded at canopy height during summer 2019 in the shade and sun treatment.

Table S1. Seed material used in the experiment.

Species	Provenance	Latitude (°)	Longitude (°)	Elevation (m a.s.l.)	Height at plantation (cm)	Diameter at plantation (mm)	Shade tolerance index*	Drought tolerance index*
Fagus sylvatica	Villorsonnens, Switzerland	46.71	6.99	740-760	49.7	5.8	4.56±0.11	2.4±0.43
Fraxinus excelsior	Lenzburg, Switzerland	47.39	8.15	400	45.8	5.1	2.66±0.13	2.5±0.25
Prunus avium	Liliental, Germany	48.08	7.68	300-600	67.4	6.3	3.33±0.33	2.66±0.22
Quercus robur	Derendingen, Switzerland	47.20	7.59	450	26.0	4.7	2.45±0.28	2.95±0.31

*tolerance scales range from 0 (no tolerance) to 5 (maximal tolerance)±SE, extracted from Niinemets and Valladares (2006).

Table S2. Amount of total carbon (C) nitrogen (N), nitrate (NO_3^-) and ammonium (NH_4^+) in the soil for each of the mesocosms at the end of the growing season (3 September 2019).

**Plot ID**	**Treatment**	**Species**	N (%)	C (%)	C:N (%)	Nitrate (mg/kg)	Ammonium (mg/kg)
1	Nutrient	Beech/oak	0.050	1.824	36.5	201.15	<0.32
2	Water	Beech/oak	0.039	1.580	40.5	<3.92	<0.32
3	Sun	Beech/oak	0.039	1.716	44.0	<3.92	<0.32
4	Drought	Beech/oak	0.039	1.619	41.5	<3.92	<0.32
5	sham drought	Beech/oak	0.037	1.689	45.6	<3.92	<0.32
6	Shade	Beech/oak	0.037	1.644	44.4	<3.92	<0.32
7	Water	Beech/oak	0.035	1.551	44.3	<3.92	<0.32
8	Nutrient	Beech/oak	0.037	1.529	41.3	<3.92	<0.32
9	Sun	Beech/oak	0.032	1.436	44.9	<3.92	<0.32
10	sham drought	Beech/oak	0.036	1.730	48.1	<3.92	0.59
11	Shade	Beech/oak	0.034	1.646	48.4	<3.92	<0.32
12	Drought	Beech/oak	0.034	1.672	49.2	<3.92	<0.32
13	Sun	Beech/oak	0.040	1.724	43.1	<3.92	<0.32
14	Nutrient	Beech/oak	0.036	1.528	42.4	90.50	8.91
15	Water	Beech/oak	0.033	1.558	47.2	<3.92	<0.32
16	Shade	Beech/oak	0.041	1.783	43.5	60.26	<0.32
17	Drought	Beech/oak	0.036	1.641	45.6	<3.92	<0.32
18	sham drought	Beech/oak	0.037	1.646	44.5	<3.92	<0.32
1	Nutrient	Cherry/ash	0.036	1.537	42.7	42.45	<0.32
2	Water	Cherry/ash	0.035	1.634	46.7	<3.92	<0.32
3	Sun	Cherry/ash	0.033	1.545	46.8	<3.92	<0.32
4	Drought	Cherry/ash	0.035	1.631	46.6	<3.92	<0.32
5	sham drought	Cherry/ash	0.033	1.672	50.7	<3.92	<0.32
6	Shade	Cherry/ash	0.036	1.619	45.0	<3.92	<0.32
7	Water	Cherry/ash	0.034	1.496	44.0	<3.92	<0.32
8	Nutrient	Cherry/ash	0.036	1.644	45.7	46.59	<0.32
9	Sun	Cherry/ash	0.035	1.630	46.6	<3.92	<0.32
10	sham drought	Cherry/ash	0.034	1.669	49.1	<3.92	<0.32
11	Shade	Cherry/ash	0.033	1.536	46.5	<3.92	<0.32
12	Drought	Cherry/ash	0.034	1.657	48.7	<3.92	<0.32
13	Sun	Cherry/ash	0.035	1.635	46.7	<3.92	<0.32
14	Nutrient	Cherry/ash	0.037	1.668	45.1	20.61	<0.32
15	Water	Cherry/ash	0.034	1.543	45.4	<3.92	<0.32
16	Shade	Cherry/ash	0.035	1.602	45.8	<3.92	<0.32
17	Drought	Cherry/ash	0.036	1.930	53.6	<3.92	<0.32
18	sham drought	Cherry/ash	0.033	1.538	46.6	<3.92	<0.32

Figure S1

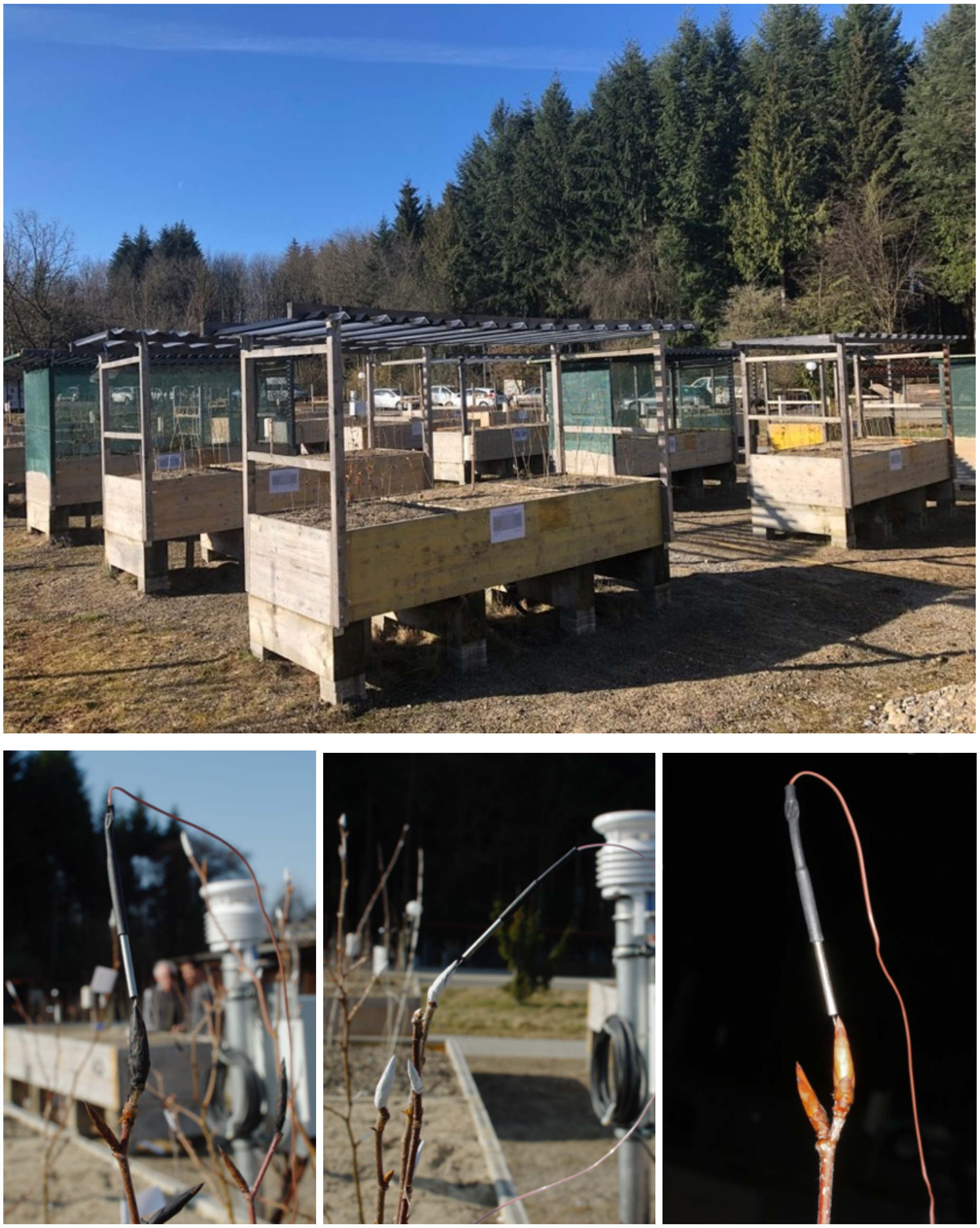

Fig. S1. Picture of the experimental infrastructure use in this study. The experimental set-up consisted of 36 containers (M1–M12) of 1 m x 1 m x 0.5 m arranged in sets of three per plot. The containers in the middle of each plot were filled with soil but did not contain any plants. On the picture is visible upfront the control-drought treatment (V-shaped plastic channels mounted downwards, covering 50% of the mesocosm surface), and towards the left and back of this plot, the drought treatment (V-shaped channels mounted upwards covering 50% of the mesocosm surface) and the shade treatment (covered from sides and top by a shading net intercepting about 70% of the incoming solar radiation). The three pictures below show white-, black- and non-painted buds of beech with the thermocouple sensor inserted into the bud to record bud temperature. Pictures credit: all from Y. Vitasse, except the last with black background from U. Wasem.

Figure S2

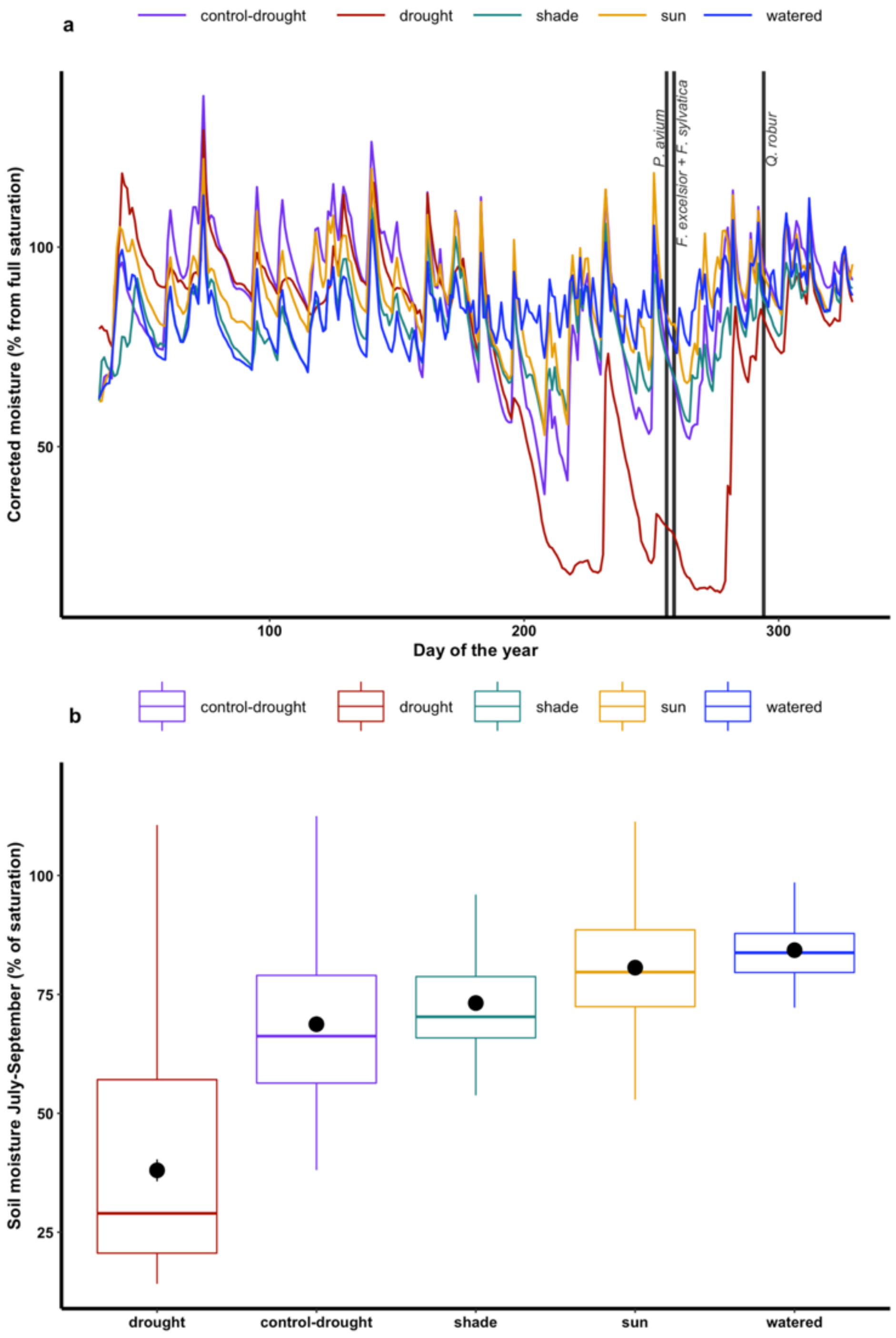

Fig. S2. Soil moisture of the different treatments shown as a percentage from full saturation during the year 2019 (a), or averaged over summer from 1 July to 30 September 2019 (b). Vertical lines in panel a represent the time when 50% leaf senescence was found for each species across all treatment. Boxplots in panel b show the first quartile, median and third quartile ± minimum and maximum values within 1.5 times the inter-quartile rang. Black dots represent the mean value of each treatment.

Figure S3

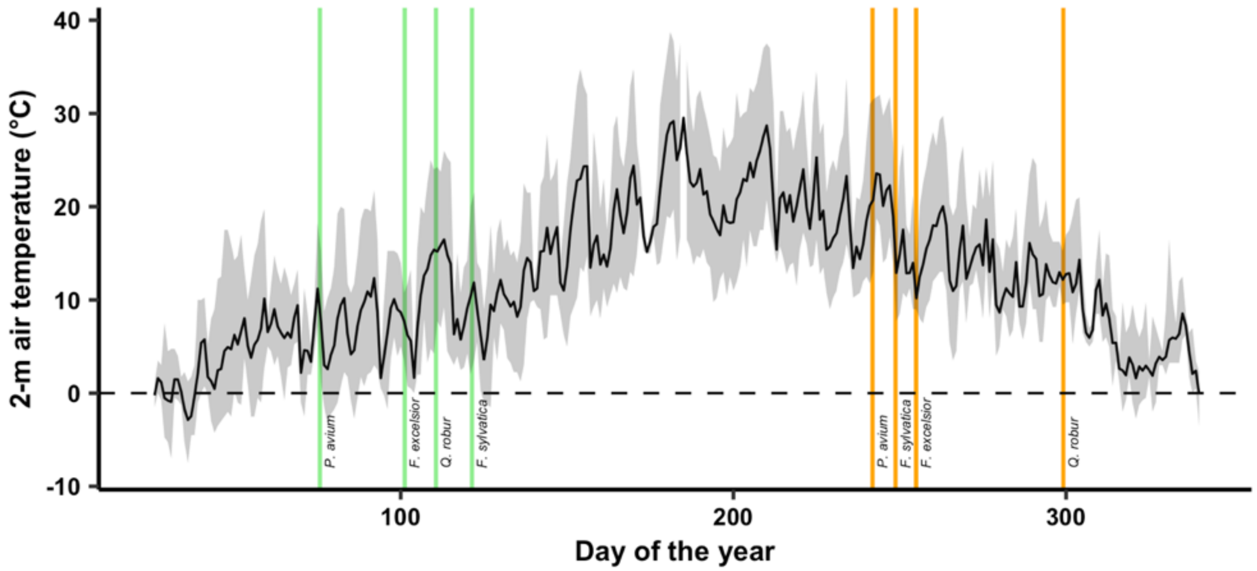

Fig. S3. Daily minimum, mean and maximum temperature recorded at 2-m height from the ground under an aluminum shelter in 2019 positioned at 2-m height. Vertical lines represent the mean date of budburst date (green) and 50% leaf senescence (orange) for each species grown within the control treatment (sun treatment). The grey area represents the range between daily minimum and maximum temperature values.

Figure S4

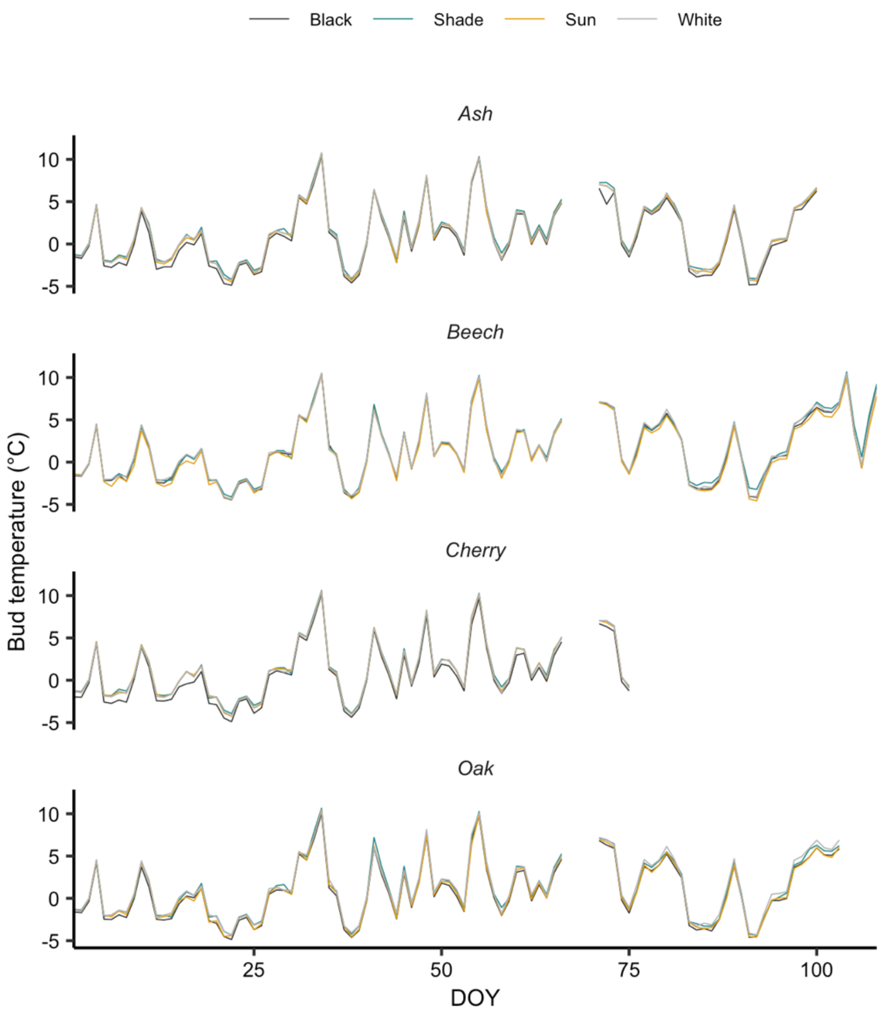

Fig. S4. Daily minimum temperatures recorded within the bud from January 2020 until species-specific budburst

Figure S5

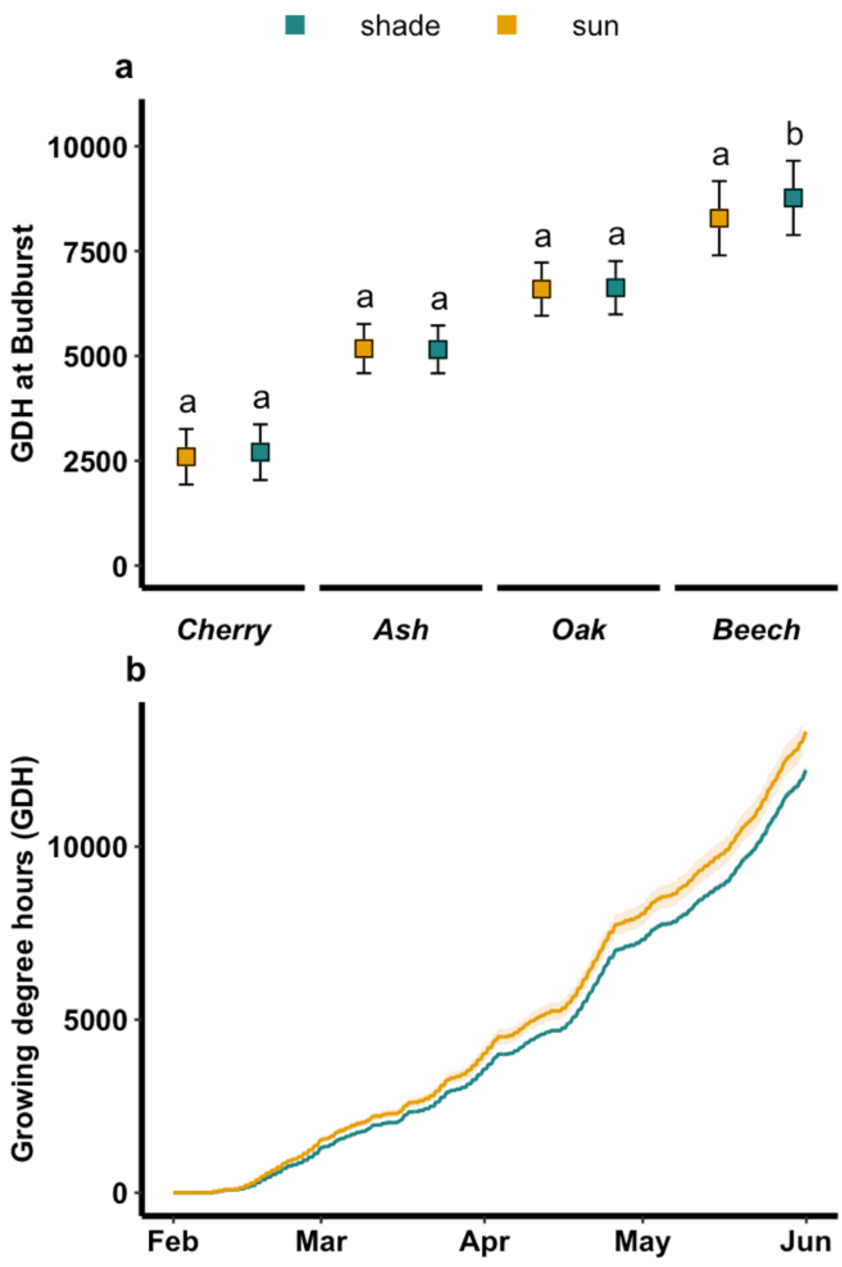

Fig. S5. Growing degree hours above 5°C recorded since 1 February 2019 until the species-specific date of budburst (a), or until end of May across species plots (b). In panel a, values correspond to the marginal mean estimates of the mixed effect ANOVA with blocks as random factor. The error bars correspond to the confidence intervals at 95%. In panel b, values correspond to the accumulation of growing degree hours surrounded by standard errors. Temperature was recorded within the plot for each species, i.e. under either the shade or sun treatment.

Figure S6

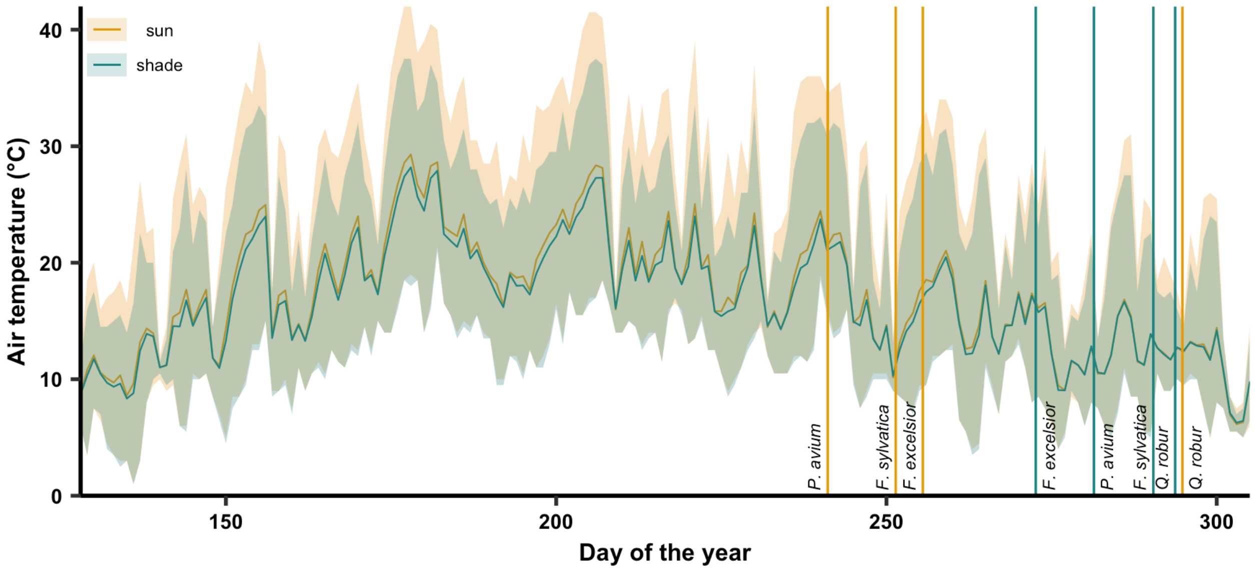

Fig. S6. Daily mean temperature surrounded by daily minimum and maximum temperature (shaded areas) recorded at canopy height in the sun and shade treatment. Vertical lines represent the mean date of 50% leaf senescence for each species grown either in the control treatment (sun treatment, yellow lines) or in the shade treatment (blue lines).

References

Niinemets U, Valladares F. 2006. Tolerance to shade, drought, and waterlogging of temperate Northern Hemisphere trees and shrubs. *Ecological Monographs* **76**(4): 521–547.
